# To Enhance or Not to Enhance? The Role of Contrast Medium ^18^F-FDG PET/CT in Recurrent Ovarian Carcinomas

**DOI:** 10.3390/medicina57060561

**Published:** 2021-06-01

**Authors:** Michela Massollo, Francesco Fiz, Gianluca Bottoni, Martina Ugolini, Francesco Paparo, Cristina Puppo, Nicoletta Provinciali, Massimiliano Iacozzi, Vania Altrinetti, Angelina Cistaro, Manlio Cabria, Andrea DeCensi, Giorgio Treglia, Arnoldo Piccardo

**Affiliations:** 1 Department of Nuclear Medicine, E.O. “Ospedali Galliera”, Mura delle Cappuccine 14, 16128 Genoa, Italy; michela.massollo@galliera.it (M.M.); gianluca.bottoni@galliera.it (G.B.); massimiliano.iacozzi@galliera.it (M.I.); vania.altrinetti@galliera.it (V.A.); angelina.cistaro@galliera.it (A.C.); manlio.cabria@galliera.it (M.C.); arnoldo.piccardo@galliera.it (A.P.); 2 IRCCS Humanitas Research Hospital, via Manzoni 56, 20089 Milan, Italy; 3 Department of Medical Physics, E.O. “Ospedali Galliera”, 16128 Genoa, Italy; martina.ugolini@galliera.it; 4 Department of Oncology, E.O. “Ospedali Galliera”, 16128 Genoa, Italy; nicoletta.provinciali@galliera.it (N.P.); andrea.decensi@galliera.it (A.D.); 5 Department of Radiology, E.O. “Ospedali Galliera, 16128 Genoa, Italy; francesco.paparo@galliera.it (F.P.); cristina.puppo@galliera.it (C.P.); 6 Faculty of Biology and Medicine, University of Lausanne, 1100 Lausanne, Switzerland; giorgio.treglia@eoc.ch; 7 Academic Education, Research and Innovation Area, General Directorate, Ente Ospedaliero Cantonale, 6500 Bellinzona, Switzerland; 8 Faculty of Biomedical Sciences, Università della Svizzera italiana, 6900 Lugano, Switzerland

**Keywords:** PET/CT, contrast enhancement, ovarian cancer, FDG, relapse

## Abstract

*Background and Objectives*: ^18^F-fluorodeoxyglucose (FDG) positron emission tomography/X-ray computed tomography (PET/CT) represents the mainstay diagnostic procedure for suspected ovarian cancer (OC) recurrence. PET/CT can be integrated with contrast medium and in various diagnostic settings; however, the effective benefit of this procedure is still debated. We aimed to compare the diagnostic capabilities of low-dose and contrast-enhanced PET/CT (PET/ldCT and PET/ceCT) in patients with suspected ovarian cancer relapse. *Materials and Methods*: 122 OC patients underwent both PET/ldCT and PET/ceCT. Two groups of nuclear medicine physicians and radiologists scored the findings as positive or negative. Clinical/radiological follow-up was used as ground truth. Sensitivity, specificity, negative/positive predictive value, and accuracy were calculated at the patient and the lesion level. *Results*: A total of 455 and 474 lesions were identified at PET/ldCT and PET/ceCT, respectively. At the lesion level, sensitivity, specificity, positive predictive value, negative predictive value, and accuracy were not significantly different between PET/ldCT and PET/ceCT (98%, 93.3%, 97.4%, 94.9%, and 96.9% for PET/ldCT; 99%, 95.5%, 98.3%, 97%, and 98% for PET/ceCT, *p* = ns). At the patient level, no significant differences in these parameters were identified (e.g., *p* = 0.22 and *p* = 0.35 for accuracy, in the peritoneum and lymph nodes, respectively). Smaller peritoneal/lymph node lesions close to physiological FDG uptake sources were found in the cases of misidentification by PET/ldCT. PET/ceCT prompted a change in clinical management in four cases (3.2%) compared to PET/ldCT. *Conclusions*: PET/ceCT does not perform better than PET/ldCT but can occasionally clarify doubtful peritoneal findings on PET/ldCT. To avoid unnecessary dose to the patient, PET/ceCT should be excluded in selected cases.

## 1. Introduction

Ovarian carcinomas (OC) represent the seventh commonest malignancy in women [1]. Even though their global incidence is relatively low, averaging 3%, they represent the first cause of gynaecological cancer death [2,3]. Moreover, treated OC has a high likelihood of relapse, which can be as high as 75%, even if there is a satisfactory response to the upfront treatment [4,5]. OC relapse is linked to its peculiar spreading mechanisms: in addition to the lymphatic and hematogenous metastastization, ovarian tumours may seed via the peritoneum, which is, in fact, the most common route of diffusion [6,7,8]. The onset of peritoneal carcinomatosis is one of the most important prognostic indicators in OC, and its detection has a key role in the planning of management protocols [9,10]. Relapse of peritoneal spread can be signalled by a rise in the tumour marker Ca-125; however, this serum protein has a poor negative predictive value and provides no information on the recurrence localization [11,12]. In opposition, imaging plays an important role in size evaluation, detection, and tumour burden assessment in peritoneal carcinomatosis, resulting in an invaluable tool in the diagnosis, able to guide therapeutic decision-making [13,14]. Among the imaging methods, the most relevant are ultrasound [15], X-ray computed tomography (CT) [16,17], magnetic resonance imaging (MRI) [18,19] and ^18^F-fluorodeoxyglucose positron emission tomography (^18^F-FDG PET/CT) [20,21]. In particular, ^18^F-FDG PET/CT outranks CT when it comes to recurrence detection and diagnostic accuracy in all body segments [22,23,24], and has shown to have an impact on patients’ management [25]. Recently the ESMO-ESGO conference advocated the use of PET/CT in the context of suspect relapse (rising tumour markers and/or clinical symptoms) [26].

Under normal circumstances, ^18^F-FDG PET/CT is performed using “low-dose” settings in the CT component: this allows performing a CT-based attenuation correction of the PET data and anatomically localizing the FDG accumulation while avoiding the increased radiation burden, which is associated with a “full-dose” CT. Some reports indicate that performing an ^18^F-FDG PET with a full-dose CT and contrast medium might grant a better accuracy than ^18^F-FDG PET with low-dose, non-contrast CT [27,28,29]. However, recent evidence was not able to confirm that using contrast medium and diagnostic settings significantly affect the accuracy of ^18^F-FDG PET/CT [30]. In general, the use of “radiological” settings and contrast medium in CT has been debated for a long time; the consensus leans toward the possible usefulness of this technique in the context of abdominal lesions [31,32].

To try and clarify the role of contrast-enhanced, full-dose PET/CT in the setting of OC recurrence, we planned this present study, in which we aimed to compare the diagnostic capability of standard “low-dose” ^18^F-FDG PET/CT (PET/ldCT) with the contrast-enhanced ^18^F-FDG PET/CT with standard radiological dose settings (PET/ceCT) retrospectively. Moreover, we tried to identify the specific clinical scenarios in which PET/ceCT could significantly outperform PET/ldCT.

## 2. Materials and Methods

### 2.1. Patient Population

The local database was searched for patients who had undergone an ^18^F-FDG PET/CT with both ceCT and ldCT in the same sitting, because of suspected recurrence of a pathologically proven ovarian cancer. At our institution, the standard imaging procedure of suspected OC recurrence is represented by an ^18^F-FDG PET/ldCT and ceCT; whenever possible, however, the two examinations are performed at the same time, to accelerate the diagnostic process and to limit the patients’ discomfort.

Indications for ^18^F-FDG PET/CT included unclear or suspicious findings at conventional imaging (CT, MRI, or ultrasound), an increase of tumour markers (at least two consecutive determinations), suggestive signs of recurrence at a physical examination, or any combination of these.

Patients were excluded in case of concomitant further oncological conditions, insufficient quality of any series of the combined examination, and in presence of ongoing treatment (including chemotherapy or external beam radiation therapy).

Each patient signed informed consent before the examination. Due to the study’s retrospective nature, the local review board waived the need to get specific consent for this study. 

### 2.2. Image Acquisition

A Discovery ST (GE Medical Systems, Milwaukee, WI, USA) PET/CT was used for the acquisition. After at least 4 h of fasting, the patients received an intravenous bolus injection of ^18^F-FDG (4.5–4.7 MBq per kilogram of body weight, in keeping with the appropriate European Association of Nuclear Medicine—EANM guidelines) [33,34]. The blood glucose levels were checked in all patients before FDG injection, and no patients showed a blood glucose level of more than 160 mg/dL. PET/CT acquisition started 60 min after that; in the meantime, the patient was hydrated orally with at least one litre of water and encouraged to void, as to diminish the background activity. PET/CT was acquired via 3-min emissions per bed position from the upper neck to the upper thighs, using sequential fields of view, each covering 12 cm (matrix of 256 × 256). PET raw data were reconstructed using ordered subset expectation maximization (OSEM, three iterations, 16 subsets), and attenuation correction was performed using the CT dataset. As per standard PET/CT imaging protocol, a 16-detector row helical CT scan was performed with low-dose current and voltage settings (120 KV, 80 mA), with a gantry rotation speed of 0.5 s and table speed of 24 mm per gantry rotation. Finally, a ceCT of the whole body (120 kV, 350 mAs, 0.5 s rotation tube), having the same axial coverage as the ldCT, was performed. The contrast-enhanced examination was performed during the portal venous phase, after intravenous injection of iodinate contrast medium (Iopamidol 370 mg/I 100 mL; with a dose of 1.5 cc/kg of patient weight, 3 mL/s). 

### 2.3. Image Analysis

All PET/ldCT examinations were grouped after anonymization, and two expert nuclear medicine physicians (MM and GB), blinded to ceCT results, interpreted the exams, with a third senior reader (AP) being referred to whenever a consensus was needed. On ^18^F-FDG PET/ldCT, any focal, non-physiological uptake higher than that of the surrounding background corresponding to any solid tissue was considered pathological [28]. Reference tissues were abdominal and pelvic lymph nodes, peritoneum, liver, lung, mediastinal, supraclavicular, and neck lymph nodes and bone.

PET/ldCT studies were interpreted visually and semi-quantitatively by using the SUVmax, on a patient-by-patient and lesion-by-lesion basis. No SUVmax cut-off values were introduced to assess tumour lesions, whereas these parameters were calculated as support to visual interpretation. All PET/ceCT results were interpreted in consensus by an experienced radiologist (FP), and by an experienced nuclear medicine physician (MC). On ^18^F-FDG PET/ceCT, any focus of non-physiological uptake corresponding to a suspicious ceCT finding was deemed suggestive for recurrence. Reference tissues were the same as described above. Enlarged lymph nodes (>10 mm) without FDG uptake were classified as negative findings [28].

### 2.4. Standard of Reference

The final diagnosis was obtained from a multidisciplinary follow-up of at least 18 months (median: 36 months, range: 18–108 months), based on tumour marker levels, contrast-enhanced CT findings, and PET/ceCT findings. We classified the findings of the cases as true positive (TP); the study revealed findings compatible with a recurrence, and the patient underwent chemotherapy resulting in a decrease in size and FDG uptake or a complete disappearance in the follow-up study. Likewise, lesions progressing under chemotherapy, or remaining initially stable under treatment but ultimately showing progression were also labelled as TP. Furthermore, lesions displaying growth in size or FDG uptake in the absence of treatment were considered TP.

True negative cases were defined as those in which no abnormal CT or PET findings were detected, and the follow-up imaging showed neither an appearance of FDG uptake nor a morphological growth or alteration of FDG-negative findings. A false positive was defined in cases in which an abnormal finding was seen at baseline imaging, with later disappearance in the absence of intercurrent treatment. Finally, the evolution in size of an FDG-negative lesion was deemed as a false negative. These criteria are summarized in Table 1.

### 2.5. Statistical Analysis

Sensitivity, specificity, and accuracy were calculated, after dichotomous interpretations of the readers (i.e., all patients and lesions were classified as negative or positive) and considering the above defined standard of reference. Differences between the two imaging modalities were tested with the McNemar. In the case of multiple comparisons, a Bonferroni correction was applied. *p* values of less than 0.05 were considered to indicate statistical significance. Statistical analyses were performed using SPSS software Advanced Models 24 (IBM, Chicago, IL, USA).

## 3. Results

### 3.1. Characteristics of the Selected Population

**Figure 1:**
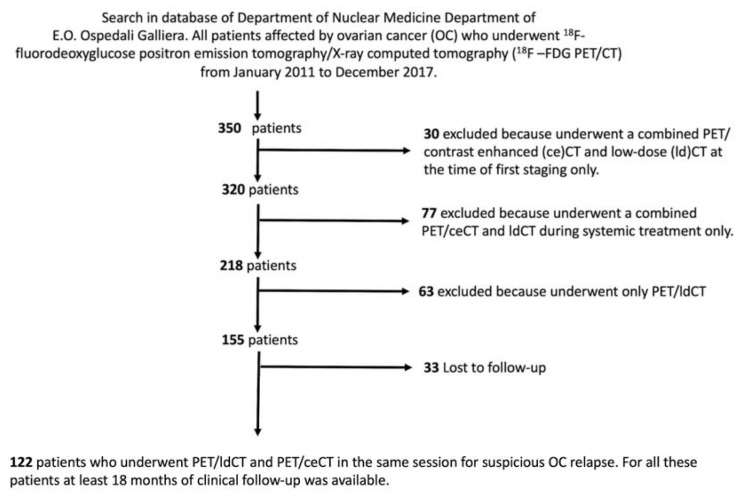
Process of patients’ selection. The flowchart details the initial size of the population sample (**top**), the number of excluded patients with relative reasons (**right**), and the final sample (**bottom**).

One hundred and twenty-two subjects (median age 57, range 27–85 years) fit the inclusion criteria (Figure 1).

Most of the cases (72%) were referred to our centres because of rising tumour marker; roughly a quarter of them had undergone a CT or MR, which had shown unconducive findings. The selected patients were affected by a generally aggressive or advanced form of OC: almost four-fifth of them had a stage III or IV at the first diagnosis, as well as a grade three clonal proliferation. The most frequent histology was serous OC, followed by the mucinous one; the other histologies made up one-fifth of the sample. Median follow-up was three years, with some patients still attending their regular follow-up visits nine years after the recurrence diagnosis. The overall patients’ characteristics are listed in Table 2.

### 3.2. Rate of Disease Identification

**Figure 2:**
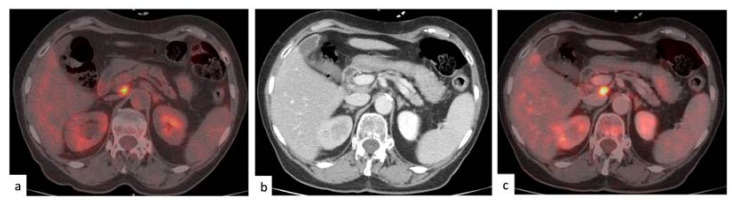
Concordant findings on positron emission tomography/low-dose computed tomography (PET/ldCT) and PET/contrast enhanced (ce)CT. An inter-aortocaval lymph node, with an intensive focal fluorodeoxyglucose (FDG) uptake, is noted on the PET/ldCT (**a**) on the ceCT part (**b**) and the PET/ceCT (**c**). Most lymph node and peritoneal lesions were classified concordantly by the two imaging methods.

A total of 56 patients (46%) had at least one finding at PET/ldCT; at PET/ceCT, 57 patients with positive findings were identified (47%). At PET/ldCT, 43 patients (35%) had at least one peritoneal lesion; among them, two had evidence of a pararectal lesion, and nine presented with a diffuse peritoneal spread. In total, 380 peritoneal lesions were identified (on average, nine per positive patient). Twenty-seven subjects (22%) had at least a positive lymph node; five had ten or more nodal manifestations. A total of 262 lesions were detected (with a mean value of 10 per positive patient). PET/ceCT detected peritoneal nodules in 44 patients (36%) and a positive lymph node in 28 subjects (23%). Overall, contrast-enhanced PET/CT identified 393 peritoneal and 263 nodal lesions. Figure 2 displays a concordant identification of a positive lymph node at both PET/CT modalities.

### 3.3. Patient-Based Analysis

Of all the 122 patients, PET/ldCT failed to identify peritoneal lesions in five patients and lymph node metastases in two. Moreover, it identified false-positive findings in the peritoneum and the lymph node stations of four and one patients, respectively. Its overall accuracy was 92.6% and 98.4% for the peritoneum and the lymph nodes, respectively. PET/ceCT performed slightly better, missing peritoneal lesions in two patients only, and lymph node lesions in one. However, the difference between the two methods did not reach statistical significance. See Table 3 for details.

### 3.4. Lesion-Based Analysis

In keeping with the patient-based setting, the lesion-based analysis could not find a significant difference in the performance of the two methods. PET/ldCT was not able to identify nine lesions (counting both peritoneal and nodal ones) while PET/ceCT had only five false negatives. Similarly, false positives lesions amounted to twelve and eight for PET/ldCT and PET/ceCT, respectively.

See Table 4 for details.

### 3.5. Change of Management Due to PET/ceCT

**Figure 3:**
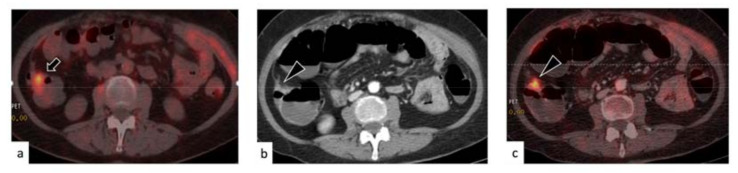
Example of clarification of a paracolic finding with positron emission tomography/contrast enhanced X-ray computed tomography (PET/ceCT). The PET/low dose (ld)CT (**a**) displayed unspecific-looking intestinal uptake, without a definite CT correlate (arrow). The PET/ceCT (**b**,**c**) identified a paracolic nodule, which was suggestive of the presence of active disease (arrowheads). This nodule disappeared after chemotherapy and therefore was confirmed as a true positive finding.

The course of therapy was altered due to the contrast-enhanced, full-dose PET/CT in four cases. In one case, a false-positive peritoneal finding in PET/ldCT was correctly characterized by PET/ceCT, and unnecessary treatment was avoided. In two cases, the presence of active disease (one lymph node and one paracolic relapse) was unmasked by PET/ceCT, whereas the unenhanced, low-dose PET/CT had not indicated any clear positivity. Because of that, treatment was initiated. The correct identification by PET/ceCT in the case of paracolic recurrence is depicted in Figure 3.

**Figure 4:**
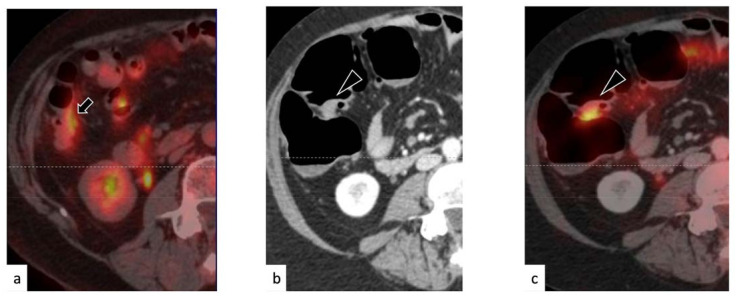
False-positive finding at positron emission tomography/contrast enhanced X-ray computed tomography PET/ceCT. The PET/ low dose (ld)CT (**a**) showed diffused, low-grade fluorodeoxyglucose (FDG) uptake within the ascending colon, which was deemed unspecific. However, the PET/ceCT (**b**,**c**) identified a seemingly nodular formation in the colic wall, with intensive FDG uptake. At further follow-up, the uptake disappeared without treatment, and the nodular formation was revealed as an artefact, due to abdominal distension.

In one case, however, PET/ceCT detected a peritoneal finding which was deemed as disease-related, even though PET/ldCT did not indicate any evident positivity (Figure 4). As the imaging was not concordant, treatment was withheld, and a control examination was scheduled in three months. The control PET/CT demonstrated a complete spontaneous disappearance of the FDG uptake; therefore, the case was classified as a PET/ceCT false positive.

## 4. Discussion

In this paper, we tried to evaluate the impact of full-dose, contrast-enhanced CT within a PET/CT examination in patients affected by OC and suspected relapse. Our results show that adding the diagnostic quality and the contrast medium has a limited impact on the method’s accuracy. The diagnostic parameters of the PET/ldCT were already very satisfactory; adding the contrast and the full dose only marginally improved these figures. However, we observed that in specific cases, a PET/ceCT could be of value. Indeed, it appears that, in selected patients with a suspected low burden of peritoneal disease, due to, e.g., marginally increase levels of tumour markers, ^18^F-FDG-PET/CeCT could be of value, allowing to correctly interpret as faint positive uptake close to the physiological activity of the bowel but corresponding to small lesions of peritoneal carcinomatosis. Besides, contrast enhancement can help the physician in identifying a small amount of abdominal and pelvic ascites whose presence often requires the physician better weigh and evaluate microscopic peritoneal lesions characterized by minimal FDG activity. Finally, using full-dose protocols can improve the grey-scale contrast and the spatial resolution in the lymph node station, thus allowing the identification of small-scale nodal disease. This consideration might be especially valid when the suspected lymph nodes are located in the proximity of physiological FDG uptake sources, such as vessels or urinary tract, where the interpretation of tracer uptake within small structures is more challenging.

In all other cases, there appears to be no compelling reason to switch to PET/ceCT. Using a full-dose setting entails a significant escalation of the absorbed dose, with a difference that can range from five- to tenfold, depending on the used protocol [35,36]. Moreover, the contrast medium has contraindications, including renal failure, which is not uncommon in ovarian cancer patients [37,38]. Finally, performing a PET/ceCT requires more time, as well as specially trained personnel. 

To date, only a few research papers have tried to tackle this issue. The study by Kitajima et al. analysed a similarly sized sample and did not detect any significant difference between PET/ldCT and PET/ceCT at the patient level [29]. At the lesion level, their data reported a slightly, albeit significantly, higher sensitivity and accuracy for PET/ceCT over PET/ldCT, even though the authors did not present cases in which the contrast-enhanced method revealed lesions undetected on PET/ldCT. Hence, it is not possible to infer from this study the specific clinical scenarios in which PET/ceCT might be considered. Of note, both PET-based methods were vastly superior, in terms of sensitivity and accuracy, when compared with ceCT. In terms of management changes, PET/ceCT affected the outcome of two per cent of patients compared to PET/ldCT. In a later report from the same group [28], PET with ldCT and ceCT in the setting of suspected OC recurrence were compared using a three-point scale (positive, equivocal, and negative). In opposition to the previous study, here Kitajima described a higher sensitivity, specificity, and accuracy of PET/ceCT at the patient level; however, the slightly different sample size might explain such a discrepancy. In particular, adding ceCT enabled the radiologist to identify the source of suspicious peritoneal nodules or lymph nodes, which were too small to be detected in the ldCT, thus increasing the physician’s confidence in categorizing the lesions as malignant. Another early report by Dirisamer et al. indicated a higher sensitivity and specificity of PET/ceCT over PET/ldCT at the peritoneal level, in a small series of patients [27]. However, a recent series analysis by Gadducci et al. could not confirm these results: both methods showed excellent sensitivity and accuracy at the patient level and performed relatively poorly in the detection of peritoneal deposits; the integration of ceCT to the PET examination brought no specific benefit [30]. In summary, although the PET/CT method and its combination with ceCT have been used for more than two decades, only a few studies performed a head-to-head comparison between the two diagnostic procedures. These data were recently summarized in a systematic review and metanalysis and did not advocate the regular use of PET/ceCT as a “one-stop-shop” in this clinical context [39].

On the other hand, there is vast evidence that FDG PET/CT has an irreplaceable role in the setting of suspected OC recurrence [40,41]. Moreover, it appears that including contrast medium administration and the diagnostic quality CT might increase the radiologist’s confidence in some selected cases. In light of this evidence, it might be advisable to integrate PET/ldCT with ceCT on a case-by-case basis, in circumstances where a marked discrepancy between PET uptake and ldCT findings are encountered, and only if this inconsistency may immediately affect the therapeutic decision-making process. In particular, the use of PET/ceCT could be encouraged in the settings where the presence of lesions amenable to surgery is suspected. In fact, relapsing OCs that can undergo secondary cytoreduction have a chance at a longer recurrence-free and overall survival, if complete resection can be achieved [42]. The use of PET/ceCT could be of use not only in identifying the disease relapse correctly but also in the correct definition of the disease extension in view of the planned surgical operation.

Moreover, protocols requiring morphometric evaluations of the disease-related findings might require having a full-dose, contrast-enhanced CT to work with; diagnostic-quality imaging is in fact preferred in the context of standardized morphologic evaluations (e.g., using RECIST 1.1) [43].

Clarification of unclear findings using PET/ceCT might be decided after the execution of PET/ldCT so that the additional exposure due to the full-dose CT ensues only when necessary. Given the available evidence, such occurrences should be relatively rare. In all other cases, given the excellent accuracy of PET/ldCT, contrast medium and diagnostic CT could be skipped, reducing the radiation burden and the patients’ discomfort.

This study had certain limitations. It represents a single-centre retrospective analysis, thus incurring potential information or selection bias. In particular, such a bias could have been introduced by a high prevalence of patients with a high disease burden. Moreover, the majority of the included population suffered from a high-grade disease, limiting our knowledge of the PET/ceCT usefulness in less aggressive ovarian neoplasms. However, these limitations are present in most studies on this subject, given the generally advanced disease status at diagnosis in OC patients [27,28]. Moreover, strict selection criteria were adopted, and all effort was put in, including only patients with complete follow-up information. As a consequence, the sample size was limited; it cannot be fully excluded that subtle differences between the two methods could have emerged by including a higher number of patients. Nonetheless, this study represents one of the largest investigations of the diagnostic power of PET/ceCT, as compared with PET/ldCT, that exist to date [39].

The ideal gold standard for any analysis would be histological confirmation of the findings. However, clinical follow-up is a valid way to evaluate diagnostic accuracy and response to therapy, and it would have been questionable to investigate all PET/CT-detected lesions using invasive procedures. Positive findings are easy to confirm, but negative findings need to be confirmed over an extended follow-up period. Therefore, sensitivity in this series may have been overestimated. 

## 5. Conclusions

Our data confirm the excellent sensitivity, specificity, and accuracy of PET/CT; moreover, they clarify the role of an integrated PET with ceCT, which might be used to clarify equivocal findings in suspected cases. However, the lack of clear indications on the superiority of PET/ceCT in terms of sensitivity, specificity, or accuracy does not support its routine use. Accordingly, the application of PET/ceCT could be decided on a case-by-case basis, according to the principle of personalized medicine.

## Figures and Tables

**Table 1:** Criteria of lesion classification.

Outcome	Definition According to Follow Up
True positive (TP)	Disappearance after treatment; Morphological increase without treatment. Progress (immediate or delayed) under therapy
True negative (TN)	No appearance of abnormal fluorodeoxyglucose (FDG) uptakes or computed tomography (CT)-evident lesions
False positive (FP)	Disappearance without treatment
False negative (FN)	Morphological increase of the FDG-negative lesion

**Table 2:** Patients’ characteristics.

**Age**	
Median	57
Range	27–85
**The Stage at Diagnosis (FIGO Staging System)**	
I	19 (16%)
II	11 (9%)
III	69 (56%)
IV	23 (19%)
**Tumor Grade**	
1	7 (6%)
2	19 (15%)
3	97 (79%)
**Tumor Histology**	
Serous	79 (65%)
Endometroid	23 (19%)
Other/nonspecified	20 (16%)
**The Main Indication for positron emission tomography/X-ray computed tomography (PET/CT) Scan**	
Rising tumor markers levels	65 (53%)
Suspect CT and/or magnetic resonance (MR) findings	19 (16%)
Rising tumor marker levels, suspicious CT and/or MR imaging findings	23(19%)
Suspicious findings at physical examination	15 (12%)
**Follow up (Months)**	
Median	36
Range	18–108

**Table 3:** Per-patient analysis of the two diagnostic methods.

	Peritoneal Lesions				
	PET/ldCT	PET/ceCT		PET/ldCT	PET/ceCT
TP	37	41	Sensitivity	88.10%	95.35%
TN	76	77	Specificity	95.00%	97.47%
FP	4	2	PPV	90.24%	95.35%
FN	5	2	NPV	93.83%	97.47%
Total	122	122	Accuracy	92.62%	96.72%
	Nodal Lesions				
	PET/ldCT	PET/ceCT		PET/ldCT	PET/ceCT
TP	26	27	Sensitivity	92.86%	96.43%
TN	94	94	Specificity	98.95%	98.95%
FP	1	1	PPV	96.30%	96.43%
FN	2	1	NPV	97.92%	98.95%
Total	122	122	Accuracy	98.36%	99.18%

Legend: TP: true positive; TN: true negative; FP: false positive; FN: false negative; PPV: positive predictive value; NPV: negative predictive value; PET/ldCT: positron emission tomography/low-dose computed tomography; ceCT: contrast-enhanced computed tomography.

**Table 4:** Per-lesion analysis of the two diagnostic methods.

	Peritoneal Lesions				
	PET/ldCT	PET/ceCT		PET/ldCT	PET/ceCT
TP	293	310	Sensitivity	97.99%	99.04%
TN	75	77	Specificity	91.46%	96.25%
FP	7	3	PPV	97.67%	99.04%
FN	6	3	NPV	92.59%	96.25%
Total	380	393	Accuracy	96.84%	98.47%
	Nodal Lesions				
	PET/ldCT	PET/ceCT		PET/ldCT	PET/ceCT
TP	162	164	Sensitivity	98.18%	98.80%
TN	92	92	Specificity	94.85%	94.85%
FP	5	5	PPV	97.01%	97.04%
FN	3	2	NPV	96.84%	97.87%
Total	262	263	Accuracy	96.95%	97.34%
	All Lesions				
	PET/ldCT	PET/ceCT		PET/ldCT	PET/ceCT
TP	455	474	Sensitivity	98.06%	98.96%
TN	167	169	Specificity	93.30%	95.48%
FP	12	8	PPV	97.43%	98.34%
FN	9	5	NPV	94.89%	97.13%
Total	642	656	Accuracy	96.88%	98.02%

Legend: TP: true positive; TN: true negative; FP: false positive; FN: false negative; PPV: positive predictive value; NPV: negative predictive value. PET/ldCT: positron emission tomography/low-dose computed tomography; ceCT: contrast-enhanced computed tomography.

## Data Availability

The data that has been used to generate the results reported in the present work can be obtained from the corresponding author, on reasonable request.
